# Properties of Al_2_O_3_/Ti/Ni Composite Obtained by Slip Casting with Different Metal Phase Content

**DOI:** 10.3390/ma15196514

**Published:** 2022-09-20

**Authors:** Marcin Wachowski, Justyna Zygmuntowicz, Robert Kosturek, Lucjan Śnieżek, Paulina Piotrkiewicz

**Affiliations:** 1 Faculty of Mechanical Engineering, Military University of Technology, 2 Gen. S. Kaliskiego Str., 00-908 Warsaw, Poland; 2 Faculty of Materials Science and Engineering, Warsaw University of Technology, Woloska 141 Str., 02-507 Warsaw, Poland

**Keywords:** composites, slip casting, Al_2_O_3_/Ti/Ni system

## Abstract

This work analyzed ceramic-metal composites from the Al_2_O_3_/Ti/Ni system produced by the slip casting method. As starting powders, nanometric Al_2_O_3_, Ni and Ti of submicron size were used. Three series of composites were obtained and tested with the same solid phase content (50% vol.) and different metallic phase content: 5, 10 and 15% vol. The influence of the metallic phase content on the basic properties of the proposed composites was examined by determining the phase composition of the produced samples using the XRD method, rheological measurements, and microstructural analysis (SEM/EDS). Additionally, before the sintering process, the slip stability was analyzed. A study of the fractography of samples after the sintering process was also carried out. It was shown that the appropriate manufacturing process allowed to obtain NiTi intermetallic phases in the structure of composites.

## 1. Introduction

Ceramic-metal composites, due to their unique properties, are an object of desire as functional and construction materials in specialized applications of electronic technology, medicine and aeronautics [1,2]. The introduction of the metallic phase dispersion into the ceramic matrix enables the shaping of a number of composite properties, such as its thermal, magnetic and electrical conductivity [3,4,5]. Considering the load-bearing capacity of this group of composites, it should be noted that the presence of plastically deformable particles gives the opportunity to dissipate energy in the loaded composite by plastic deformation of the metallic phase without losing the cohesion of the ceramic matrix [6]. Both the possibility of modifying the physicochemical properties and increasing the strength parameters of composites create new opportunities for the development of innovative materials operating in high temperature, friction or corrosive environments [7,8,9]. In the literature, few ceramic-metal composites are widely described in terms of welding and rolling of Ti-15Mo/TiB [10,11], microstructure and properties of TiBw/Ti6Al4V [12,13], and manufacturing and characterization of Ti-TiB composites [14,15,16,17]. It is worth mentioning that a significant modification of this group of composites may be the replacement of metallic phases with intermetallic phases, which in their properties constitute a kind of bridge between metallic and ceramic materials [18,19]. The combination of ductility, high strength, abrasion resistance and hardness makes the intermetallic compounds an excellent modification of the classic, metallic reinforcement of ceramic composites [20].

The main problem is to develop an effective technology for obtaining reinforcement with the intermetallic phase while maintaining an even distribution of particles in the ceramic matrix. The production of the composite can be accomplished both by introducing intermetallic particles into the ceramic mass and by a mixture of two metal particles capable of forming them, and then by creating intermetallic phases by heat treatment [21]. Heat treatment does not have to constitute an additional stage of production as a post-processing of the composite, but it can be carried out as a result of the influence of temperature in the sintering process of the ceramic matrix, making it possible to obtain the finished product in a relatively low complex technological process [22]. In the latter variant, mixed reinforcement consisting of both metallic and intermetallic particles can be obtained. A number of research studies have already shown that intermetallic compounds significantly improve the fracture toughness and bending and compressive strength of ceramic matrix composites [23,24].

Some of the most popular ceramic composites are those based on the Al_2_O_3_ matrix, among the main advantages of which are high strength parameters and low production costs [25,26]. When considering potential intermetallic reinforcements, the two compounds from the Ni-Ti system: NiTi and Ni_3_Ti deserve special attention. They are characterized by high strength parameters, including hardness and resistance to abrasion at elevated temperatures, thus constituting a potential strengthening phase for ceramic composites dedicated to high-temperature applications [27]. In the formation of Ni-Ti intermetallic compounds, the NiTi_2_ phase should be avoided due to its low mechanical properties often causing a failure in its layer [28]. The paper is a continuation of research previously published in paper [29], in which the influence of different solid phase content (35%, 50%) on microstructure, mechanical properties, and possibilities of manufacturing of Al_2_O_3_/Ti/Ni composites with intermetallic phases was investigated. In all previously investigated composites 15% vol metallic phase content was constant. Previous studies have shown that the use of a mixture of metallic nickel and titanium powders in the production of the Al_2_O_3_ matrix enables the production of NiTi and Ni_3_Ti intermetallic compounds, significantly increasing the strength parameters of the composite [29]. The process of obtaining such a composite can be successfully carried out by slip casting and sintering [30]. According to previously presented research results, in this study, Al_2_O_3_/Ti/Ni composites were obtained and tested with the same solid phase content (50%) and different metallic phase content: 5, 10 and 15% vol.

This paper presents the results of research on the preparation of an Al_2_O_3_/Ni/Ti composite reinforced with intermetallic phases with a different proportion of the metallic phase, amounting to 5, 10 and 15% (the ratio of metallic components 1:1) and the solid phase content equal to 50% vol. The use of a finer metal powder allowed for a significant increase in the share of intermetallic phases in relation to the previous studies [29]. The first stage of work focused on characterizing the starting materials. Then, the slip materials used to form the samples were assessed. In the next stage, tests were carried out to characterize the basic properties of the proposed composite, including phase analysis and microstructure observations, as well as EDS testing.

## 2. Materials and Methods

The starting materials were: alumina powder under the trade name TM-DAR by Tamei Chemicals Co., (Nagano, Japan), titanium powder from GoodFellow Cambridge Limited (Huntingdon, UK) and nickel powder from Sigma-Aldrich (St. Louis, MO, USA). The specifications of the powders provided by the manufacturers are presented in Table 1.

This study decided to use aluminum oxide in the form of α because it is characterized by a very high melting point (2051 °C) and high specific resistance. In addition, this compound is also characterized by high stability and chemical resistance. It is resistant to organic and inorganic acid solutions as well as to most concentrated acids at room temperature. In industry, aluminum oxide is widely used in applications requiring chemical resistance, thermal shock resistance and electrical resistance. It is used as a material for cutting and grinding tools, fire protection and others. Due to a number of advantages of Al_2_O_3_, such as the aforementioned chemical and thermal resistance, or high hardness, it is a commonly used material in materials engineering, both in the production of solid ceramic elements and as a matrix in ceramic-metal composites. Alumina particles are also used, among others, in metal matrix composites as a material reinforcing phase. On the other hand, nickel has good corrosion resistance in seawater, mineral waters and organic acids. It reacts in the environment of phosphoric and nitric acid or sulphur compounds. It belongs to the group of elements that are characterized by ferromagnetic properties at room temperature (below the Curie point—temp. 358 °C). Nickel is used in the form of coatings to increase strength and corrosion resistance, while nickel-based superalloys are used in the energy industry for heat resistance and creep resistance at high temperatures. In turn, titanium is a relatively light metal (4.5 g/cm^3^) with high mechanical properties. Due to its attractive physical, mechanical and chemical properties, it has found wide application in various fields of engineering. However, in the case of Al_2_O_3_-metal composites, the use of titanium alone as an additional phase will not significantly improve the mechanical properties due to its low hardness. The improvement of the properties of the Al_2_O_3_/Ti composite can be achieved by obtaining an additional TiO_2_ phase in the composite, using an appropriate sintering process and atmosphere, or by using an additional metallic material. Titanium in a properly selected homogenization and sintering process with nickel as the second metal component will enable the formation of TiNi and/or TiNi_3_ intermetallic phases in the composite, which will significantly improve the final properties of the product. In recent years, there has been a lot of interest in materials containing the TiNi phase due to its properties. The TiNi phase is characterized by good biocompatibility, excellent corrosion resistance and shape memory effect. TiNi materials and composites containing this phase are used as elements in electromechanical systems, elements of sensors for actuators and medical implants. The NiTi phase and the preceding Ni_3_Ti and NiTi_2_ phases show both high hardness and wear resistance.

Measurements of the specific (real) density of the Al_2_O_3_, Ni, Ti powders were carried out using the Accu Puc II automatic gas pycnometer by Micrometrics (Norcross, GA, USA). Measurements were carried out in a cylindrical measuring chamber 19 mm in diameter × 39.8 mm in height. The pressure of the measurement chamber filled with helium: 19.5 psig. measurement accuracy: 0.03%, measurement repeatability: ±0.01%. The powders were tested with parameters of 700 cycles and 10 washes in the presence of helium.

For the research in this work, the Rigaku Mini Flex II diffractogram (Tokyo, Japan) with CuKα radiation and wavelength of λ = 1.5406 Å was used, the following parameters were applied: angular range 2θ—20–100°, voltage—30 kV, current—15 mA, measurement step —0.02°, counting time—2 s. The research was carried out in order to characterize the phases of the starting materials. Moreover, the phase composition of the shapes in the raw state and after the sintering process was determined.

Composite samples from the Al_2_O_3_/Ti/Ni system were made by slip casting. The production process of Al_2_O_3_/Ti/Ni composites was carried out according to the following procedure:

- Weighing out the correct amount of powders, liquefier and water;
- Combining and mixing the weighed ingredients to harmonize the consistency;
- Homogenization and deaeration of the mixture.

Homogenization was carried out in two stages. In the first stage, the prepared suspension was homogenized in a planetary-ball mill PM 400 (Retsch, Haan, Germany), which is a floor model with 4 grinding stations. The process was carried out for 1 h with a rotational speed of 300 r/min. During homogenization, the vessels with suspensions were placed off-centre on the rotating base of the planetary-ball mill. The direction of rotation of the base was opposite to that of the vessels and the speed ratio wass 1:2. The movement of the balls inside the vessel is the result of the so-called Coriolis forces. The different velocities between the balls and the vessel lead to the interaction of the frictional and impact forces, which generate high dynamic energy. The interaction of these two phenomena leads to the achievement of a very high degree of grinding the ground metal. Then, in the second stage, the slip prepared in this way is placed in the high-speed homogenizer THINKY ARE-250 (Tokyo, Japan), where the further homogenization process is carried out. The first stage of homogenization in the THINKY ARE-250 device is mixing for 8 min with a rotational speed of 1000 r/min, and then deaeration of the mixture for 2 min with a rotational speed of 2000 r/min.

- Pouring the deaerated mixture into previously prepared cylindrical plaster moulds with a diameter of 20 mm and a height of 10 mm;
- Sample drying process;

The samples were dried in a laboratory dryer (Carbolite·Gero, Neuhausen, Germany)at 40 °C for 48 h.

- Mechanical processing.

The samples were ground on sandpaper with the following grades: 120, 400, 600, 800 and 1200 in order to obtain a shape with flat-parallel surfaces.

Modifying agents were used for the preparation of the slip, which were used for the purpose of proper liquefaction, thus giving the required rheological properties to the slip. A composition of liquefiers was used, which was citric acid (CA) and diammonium citrate (DAC). In the further part of the work, it was assumed to denote citric acid as CA and diammonium citrate as DAC.

One of the simplest methods of analyzing the stability of the suspension is to conduct macroscopic observations as a function of time from its production. In a properly composed slip, sedimentation does not take place or it takes longer than in the case of a suspension without fluidizing and stabilizing additives. In this study, three suspensions from the Al_2_O_3_/Ti/Ni system with 50% vol. of the content of the solid phase and various contents of the metallic phase were prepared (5%, 10% and 15% volume of the metallic phase)—Table 2. During the observation, containers with suspensions were photographed at specified intervals from moulding for 1 h in order to determine the tendency to sedimentation.

Microstructural observations of the starting powders and sintered fittings were made using the JEOL JSM-6610 (Tokyo, Japan) scanning electron microscope (SEM) with the use of a secondary electron (SE) detector. The observation was made at an applied accelerating voltage of 15 kV. Due to the low conductivity of the material, additional metallization of the sample was necessary. A carbon target was used for metallization.

The rheological measurements were used to study the rheological properties of slippery masses, such as viscosity, thixotropy and to determine the flow curves. The measurement allowed us to determine the appropriate technical or economic parameters of the mass production, forming and drying processes of ceramic semi-finished products. The obtained results are shown in the diagram of the flow curve, which is the dependence of the shear rate as a function of shear stress, and also in the diagram of the viscosity curve, which is the dependence of the shear rate in the function of fluid viscosity. The tested rheological properties were measured using a DV3T rotary rheometer (AMETEK GmbH/B.U. Brookfield, Hadamar-Steinbach, Germany) with a DIN-87 spindle and the test parameters:

- number of revolutions per minute: 25;
- initial speed: 0.8 revolutions/min;
- final speed: 25 revolutions/min;
- holding time for each speed: 10 s;
- temperature: 25 °C.

Slip casting is one of the basic methods of producing ceramic samples and ceramics-ceramics or ceramics-metal composites. The starting material in this method are powders of individual components, which, as a result of an appropriately optimized production process, with the participation of fluidizers and a solvent, are used to produce solid samples. There are many modifications to this production method; samples can be cast on flat surfaces, into appropriate moulds of various shapes, or by additionally using centrifugal casting. Regardless of the casting method chosen, porous plaster substrates/moulds are used in the process. The plaster, thanks to the action of capillary forces, draws the solvent (water) from the moulded slip, making it possible to obtain solid samples. The process of making plaster moulds begins with weighing out water and plaster in appropriate predetermined proportions in separate containers. Then the plaster is mixed with water, and the resulting mixture, after thorough mixing, is filtered into a separate container to remove any agglomerates. The hydrated plaster is then poured into a properly prepared matrix, which is responsible for mapping the appropriate shape of the mould. The process uses matrices made of epoxy resin. In the next stage, the plaster poured into the mould is placed on a shaker in order to remove the air bubbles inside for about 1 min. The prepared mass is left to bind. After the plaster binder has hardened, the produced plaster mould is separated from the mother mould and placed in a laboratory drier until completely dry (48 h, 30 °C).

The sintering process of composite fittings produced by the centrifugal casting method was carried out in the Nabertherm RFTC 80230/16 furnace (Nabertherm GmbH, Lilienthal, Germany). It is a compact tube furnace equipped with SiC heating rods and an integrated switchgear with a controller. SiC heating rods installed parallel to the working tube ensure perfect temperature uniformity. The entire sintering process took 24 h in a reducing atmosphere with 95% argon and 5% hydrogen content. In the first stage, from a temperature of 23 °C to 120 °C, the samples were heated with a heating speed of 5 °C/min, in the second stage from a temperature of 120 °C to 750 °C with a heating speed of 1 °C/min, and in the last stage from a temperature of 750 °C with a heating rate of 2 °C/min, until the holding temperature of 1400 °C. The samples were kept at the temperature of 1400 °C for 2 h, then the samples were cooled with a cooling speed of 6 °C/min.

## 3. Results and Discussion

In the first stage of the research, the actual density of aluminum, nickel and titanium oxide powders was determined using the pycnometric method, taking into account the non-zero volume of the measuring vessel. Table 3 presents the obtained results. The studies showed that the determined real densities of the Al_2_O_3_ and Ti powders are the same as the theoretical density declared by the manufacturer. Taking into account the measurement error of the pycnometric method, it was found that the determined real density of the nickel powders was lower than the theoretical density declared by the manufacturer. It may be caused by slight surface oxidation of the powder.

**Figure 1:**
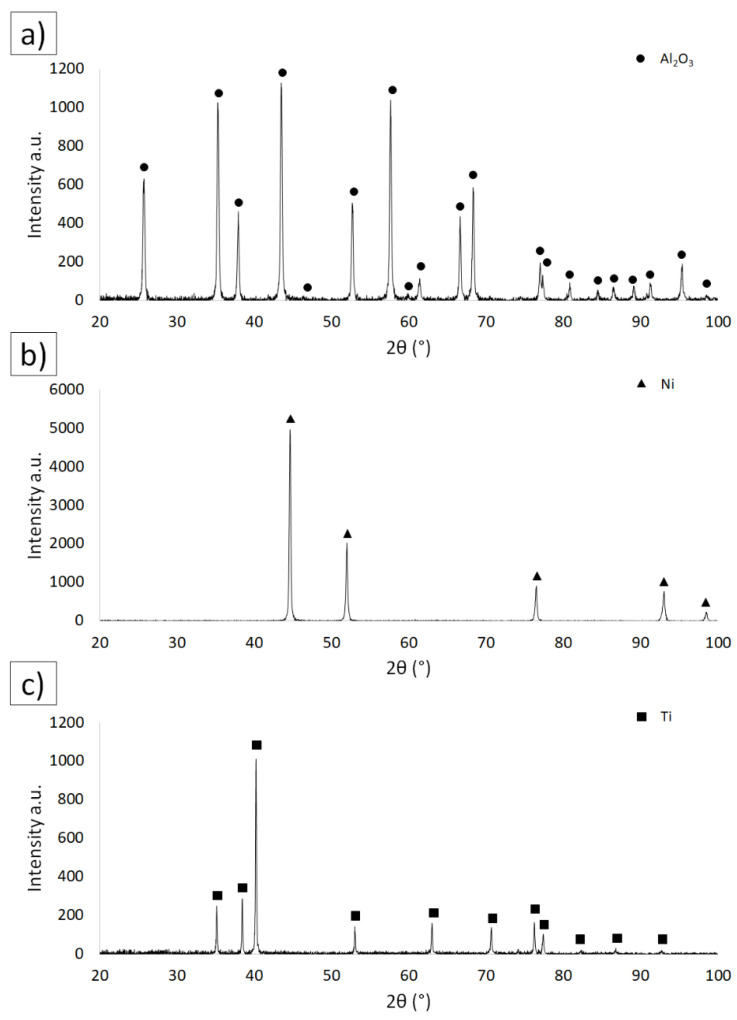
Diffractograms of the starting powders: (**a**) Al_2_O_3_, (**b**) Ni and (**c**) Ti.

Figure 1 shows the results of the phase analysis for aluminum oxide (Figure 1a), nickel (Figure 1b) and titanium (Figure 1b) powders. The analysis of the obtained results for alumina was carried out on the basis of the safety data sheets with the number PDF #04-003-7263, for nickel on the basis of the cards with the number PDF #04-003-7263, and for titanium on the basis of the cards with the number PDF #01-083-5019. Based on the analysis of the obtained diffractogram, it can be noticed that there are no peaks coming from other phases, which confirms the high purity of the powders declared by the manufacturers.

**Figure 2:**
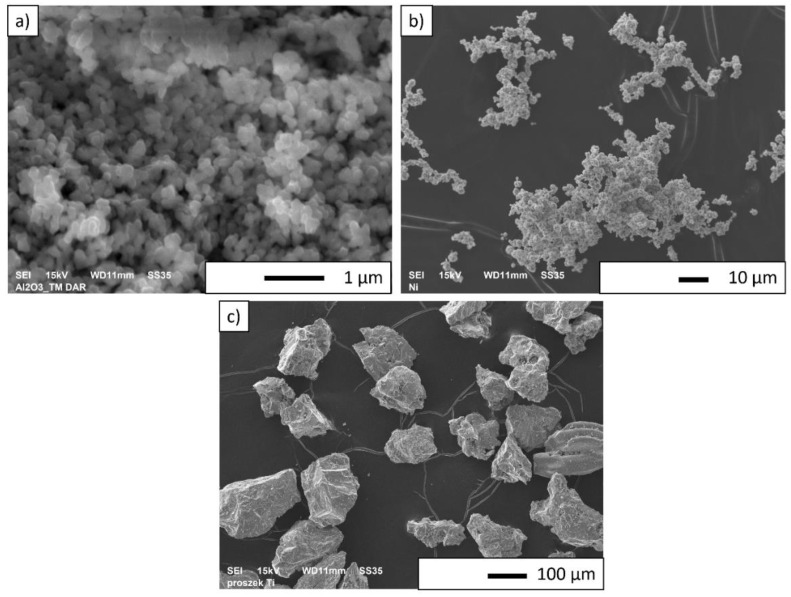
Sample SEM photos of Al_2_O_3_ (**a**), Ni (**b**) and Ti (**c**) powder.

In the next step, microscopic observations of alumina powder were carried out. Figure 2 shows sample SEM pictures of Al_2_O_3_ (a), Ni (b) and Ti (c) powders. The analysis of the obtained photos showed that the alumina powder has a tendency to form agglomerates. Moreover, when analyzing the presented photomicrographs, it can be noticed that the Al_2_O_3_ powder is characterized by grains of slightly different shapes. It was found that the nickel powder particles have a dendritic shape with irregular morphology, while the titanium powder particles have a flake shape.

**Figure 3:**
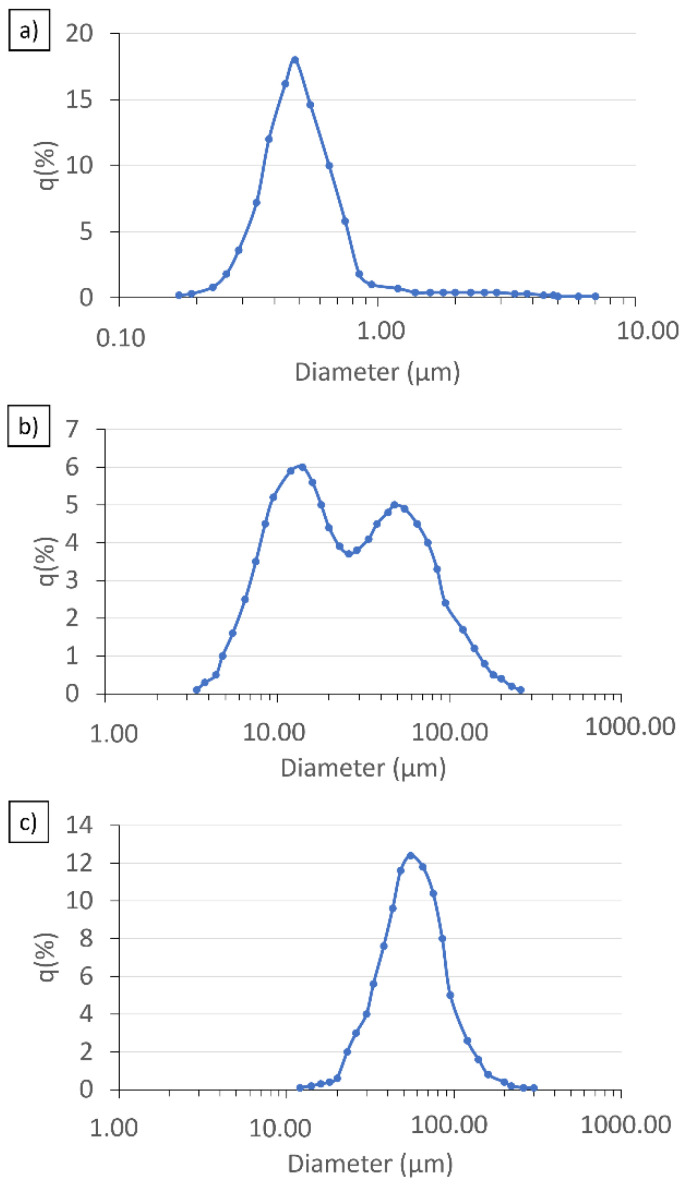
Results generated for aluminum (**a**), nickel (**b**) and titanium (**c**) oxide powders by dynamic light scattering.

Then, as a result of laser particle size measurement, a distribution was obtained, which represents the volume percentage of individual fractions with a given particle diameter of Al_2_O_3_, Ni and Ti in the total volume of powders. Figure 3a–c show the results obtained. The average particle size of the alumina powder (Figure 3a) obtained during the test is equal to 0.61 µm, and the median is 0.48 µm. The particle diameter range of PSD distribution is very wide, ranging from 0.17 µm to 7.69 µm. The most abundant fraction of 17.24% of the powder volume are particles with a diameter of 0.51 µm. The obtained results differ from the average particle size declared by the manufacturer. This is due to the fact that alumina powder is prone to form an agglomerate formation, which has been confirmed by SEM observations. Figure 3b shows the results for the nickel powder. The mean particle size of the nickel powder obtained during the test is 35.888 µm and the median 22.86 µm. The particle diameter range of the PSD distribution is bimodal and ranges from 3.41 µm to 262.38 µm. The analysis revealed the presence of two powder fractions ranging from 3.409 µm to 26.11 µm and from 26.11 µm to 262.38 µm. In the case of the first fraction, the particles with a diameter of 13.25 µm represent the largest batch of 6.005% of the powder volume. In the case of the second fraction, the largest batch of 4.98% of the powder volume are particles with a diameter of 51.47 µm. The obtained results differ slightly from the mean particle size declared by the manufacturer < 50 µm. The test shows that the average particle size of the nickel particles is about 35 µm, however, there are also particles larger than 50 µm. Figure 3c shows the results for the titanium powder. The mean particle size of the titanium powder obtained during the test is 57.89 µm, and the median is 53.46 µm. The particle diameter range of the PSD distribution is wide, ranging from 11.57 µm to 262.38 µm. The most abundant fraction of 12.40% of the powder volume are particles with a diameter of 58.95 µm. 

**Figure 4:**
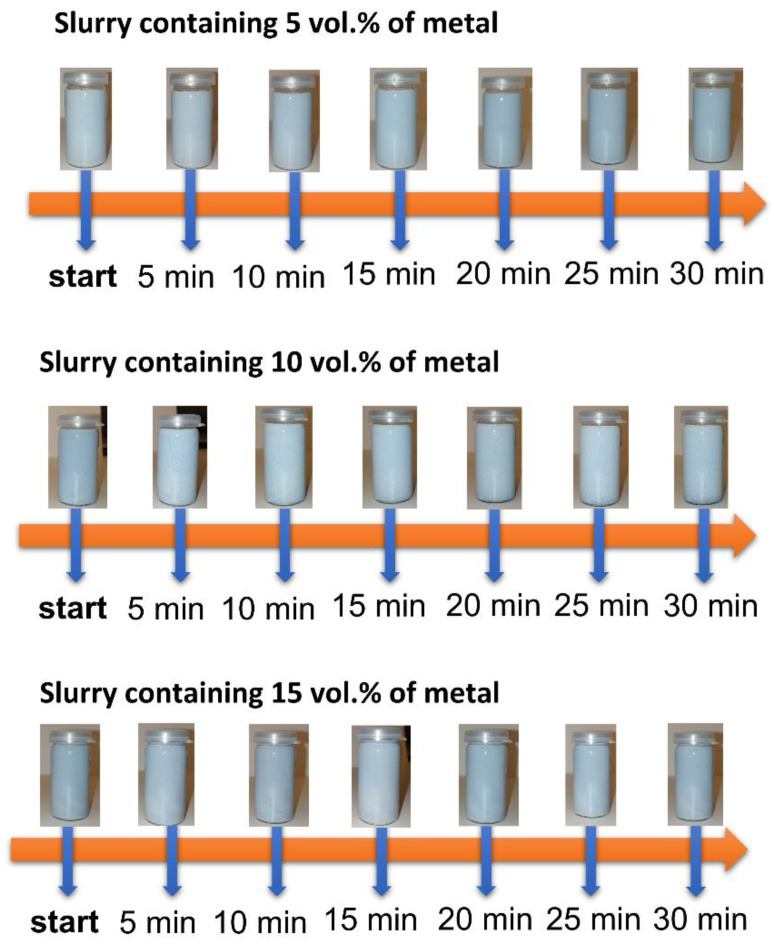
Macroscopic observations of slippery masses over time.

Three Al_2_O_3_/Ti/Ni suspensions were prepared with 50% volume of the solid phase content and different content of the metallic phase (5%, 10% and 15% of the volume of the metallic phase). During the observation, containers with suspensions were photographed at specified time intervals from pouring through 30 min. Figure 4 shows the photos of the slippery mass over time. As can be seen, the color of the slips differed slightly, which results from the different content of the metallic phase in the prepared suspensions. The higher the content of metallic particles, the more intense the color of the obtained slurry is. All the slips were homogenous after casting. The occurrence of delamination, uneven proportion of the metallic phase or sedimentation was not observed. The observation of the slips did not reveal any changes in the appearance of the slip in the first 30 min from the moment of casting. The lack of tendency of the obtained suspensions to sedimentation at this time allows the conclusion that they were properly composed and can be used for the production of composite samples from the Al_2_O_3_/Ti/Ni sample. After 30 min, slight sedimentation was observed at the bottom of the dishes. In the case of moulding fittings, the slip casting method is of key importance in the first 15–20 min from the moment of preparing the suspension. This time is necessary to properly pour the suspension into the plaster mould.

**Figure 5:**
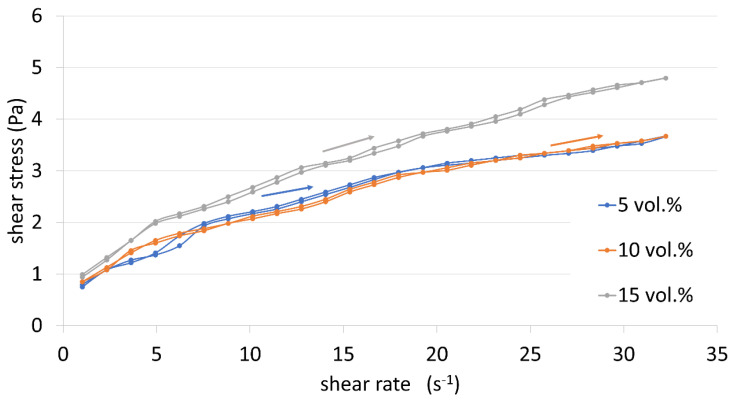
Suspension flow curves.

**Figure 6:**
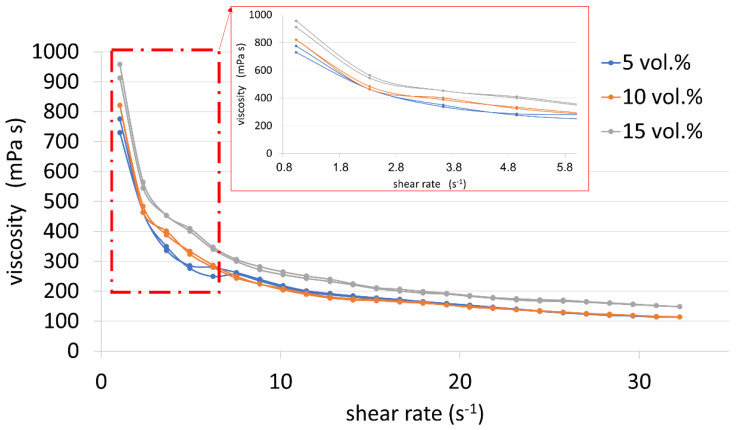
Viscosity curves of suspensions.

The next step was to focus on rheological research. The obtained results for the suspensions were presented in the form of collective diagrams showing the flow curves (Figure 5) and the viscosity curves (Figure 6). Based on the flow curves shown in Figure 5, it can be concluded that all suspensions are thixotropic fluids, as evidenced by the hysteresis loops visible in the curves. Moreover, all the suspensions have low yield points, less than 1.5 Pa.

Based on the course of the viscosity curves (Figure 6), it can be concluded that all analyzed suspensions are shear-thinning fluids, because their viscosity decreases with increasing shear rate. Moreover, it was observed that increasing the content of the metallic phase causes an increase in the viscosity of the tested slip. The viscosity values at the shear rate of approx. 1.032 s^-1^ are respectively 775.6 mPa∙s, 821.2 mPa∙s and 912.5 mPa∙s for suspensions with the proportion of the metallic phase amounting to 5% vol., 10% vol. and 15% vol. These are preferably low viscosity values that allow the process of forming the suspension to be carried out correctly.

**Figure 7:**
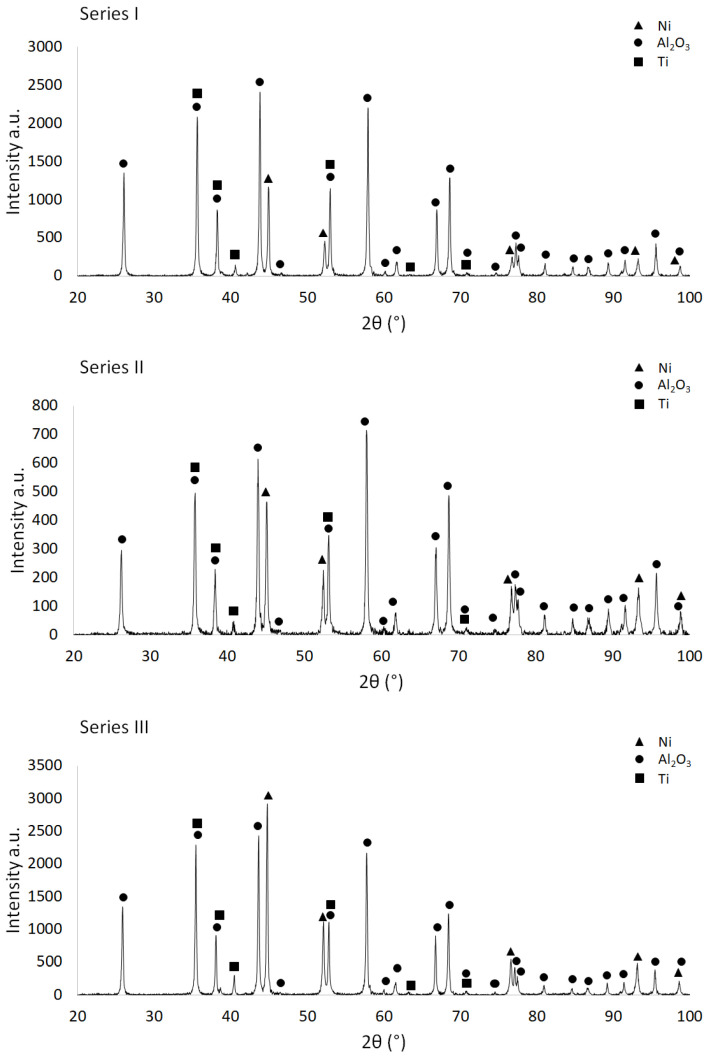
Diffractograms for raw samples.

Based on macroscopic observations of the unfinished fittings, it was found that the obtained composites were characterized by the absence of visible defects in the form of cracks, microcracks and delamination on the surface. The phase composition of the produced samples was then determined. Figure 7 shows the diffractograms obtained for samples in the raw state. In all series of raw samples (Figure 7), regardless of the content of the metallic phase in the composite structure, the phase composition test confirmed the presence of three phases: hexagonal Al_2_O_3_ (PDF #04-003-7263) and Ti (PDF #01-083-5019), as well as cubic Ni (PDF #04-003-7263). Direct measurements made it possible to determine characteristic spectral curves for composite samples with different contents of the metallic phase. In the case of Series I in the raw state, containing 5% vol. of the metallic phase in the structure, the reflections observed for the values of the angle 2° equal to, respectively: 44.96°, 52.32°, 76.81°, 93.30° and 98.72° correspond to the following Ni crystallographic planes: (111), (200), (220), (311) and (222). For Series II samples, 10% vol. of the content of the metallic phase, reflections from the same crystallographic planes were observed at the values of the 2° angle equal to, respectively: 45.08°, 52.42°, 76.82°, 93.36° and 98.79°. For Series III, containing 15% vol. of the metallic phase in the structure, the values of the 2° angle to which the characteristic Ni crystallographic planes were assigned were, respectively: 44.80°, 52.14°, 76.64°, 93.16° and 98.45°. In the case of titanium, the Series I diffractogram analysis revealed the presence of reflections from the following crystallographic planes: (100), (002), (101), (102), (110) and (103), with the 2° angle values, respectively: 35.64°, 38.82°, 40.63°, 53.04°, 63.41° and 70.84°. Identical reflections from the above-mentioned crystallographic planes were also observed in the Series II and Series III diffractograms. In the case of Series II, they occurred at the values of the angle 2° equal to, respectively: 35.79°, 38.36°, 40.67°, 53.12°, 61.84° and 70.84°. For Series III, the values of the 2° angle, at which the presence of characteristic reflections from the Ti crystallographic planes were found, were equal to, respectively: 35.48°, 38.70°, 40.50°, 52.14°, 61.58° and 70.84°.

**Figure 8:**
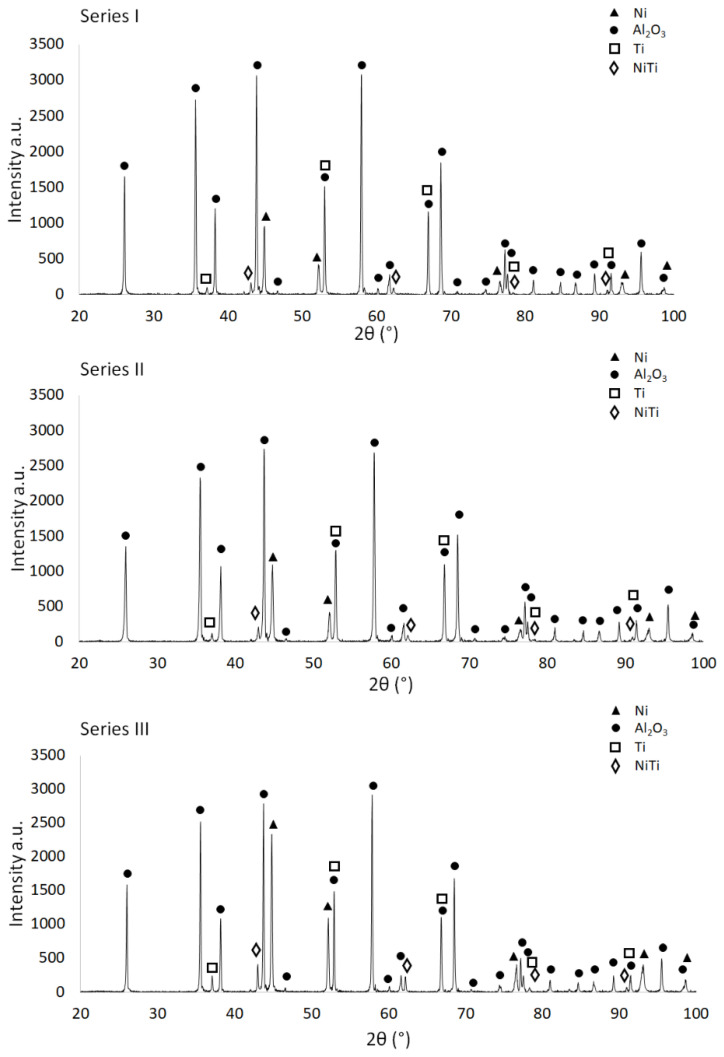
Diffractograms for samples after the sintering process.

Analysis of the phase composition of the same samples of individual series after the sintering process (Figure 8) revealed the presence of an additional NiTi phase in all three series (PDF #00-018-0899). In addition to the diffraction patterns of the raw hexagonal Al_2_O_3_ and cubic Ni shapes previously observed in the diffraction patterns, the samples after sintering showed the presence of cubic Ti in the structure (PDF #04-003-7272). In all analyzed series, regardless of the content of the metallic phase, XRD revealed the presence of reflections coming from the planes characteristic for cubic Ti: (110), (200), (211), (220). In Series I diffraction patterns, reflections from these planes were observed for the values of the angle 2° equal to: 38.43°, 52.86°, 66.78° and 78.22°. Similar values of the angle 2° for these crystallographic planes were observed for Series II (respectively, 36.94°, 53.14°, 66.28° and 78.16°) and Series III (in this case, respectively, 36.84°, 52.73°, 66.66° and 78.22°). The presence of Ti with a different crystallographic structure clearly indicates the phase transformation of this component taking place during the sintering process. In the sintered samples, regardless of the metallic phase content in the structure, the diffraction spectrum showed the presence of reflections from the same Ni crystallographic planes as in the raw samples, namely (111), (200), (220), (311), with the values of the angle 2° equal to, respectively: 42.94°, 52.08°, 76.60°, 93.08° and 98.64° for Series I, while for Series II the values were, respectively: 44.62°, 51.90°, 76.42°, 92.90° and 98.46°. In the case of Series III, successive reflections from the above-mentioned Ni crystallographic planes were observed at the 2° angle values equal to 44.70°, 52.04°, 76.50°, 92.96° and 98.46°. The new NiTi phase formed during the sintering process was visible in the diffraction patterns of the sintered samples of all series in the form of three characteristic reflections coming from the crystallographic planes (110), (200) and (211). In Series I samples with the lowest metallic phase content, reflections from these planes were observed at the 2° angle values equal to 42.94°, 62.12° and 78.22°. In the case of Series II and Series III, reflections from subsequent planes occurred at the angle values of 2° equal to, respectively: 42.81°, 61.95° and 77.96° for Series II, while for Series III they were analogically equal to 42.87°, 62.04° and 78.16°.

The formation of new phases as a result of the sintering of samples from the Al_2_O_3_/Ti/Ni system is confirmed by the available literature on the subject, which clearly indicates the potential of intermetallic formation in composites containing both Ti and Ni in the structure [31]. The presence of intermetallic phases in the produced composites may be caused by the fact that the addition of nickel to titanium alloys and, at the same time, other alloying elements stabilizes the β phase with a shift in the eutectoid transformation β => α towards low temperatures [13]. In combination with titanium, nickel stabilizes the β phase and creates many intermetallic phases, of which three are very well known: TiNi, Ti_2_Ni and TiNi_3_ [32]. This is also confirmed by the results of previous research carried out by the team, where also, in Al_2_O_3_/Ti/Ni composites after the sintering process, XRD analysis revealed the presence of new intermetallic phases [33]. The fact that the metallic powders did not react completely relates to the structure of the composite. To form an intermetallic compound, the particles of Ni and Ti must be in direct contact with each other. In the obtained composite, a large part of the Ni and Ti particles is separated by the Al_2_O_3_ matrix inhibiting Ni-Ti diffusion.

**Figure 9:**
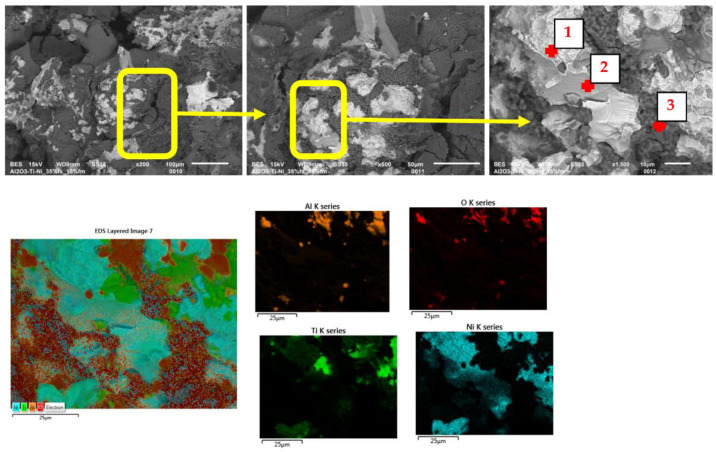
SEM/EDS results of samples with 5% volume of the metallic phase.

**Figure 10:**
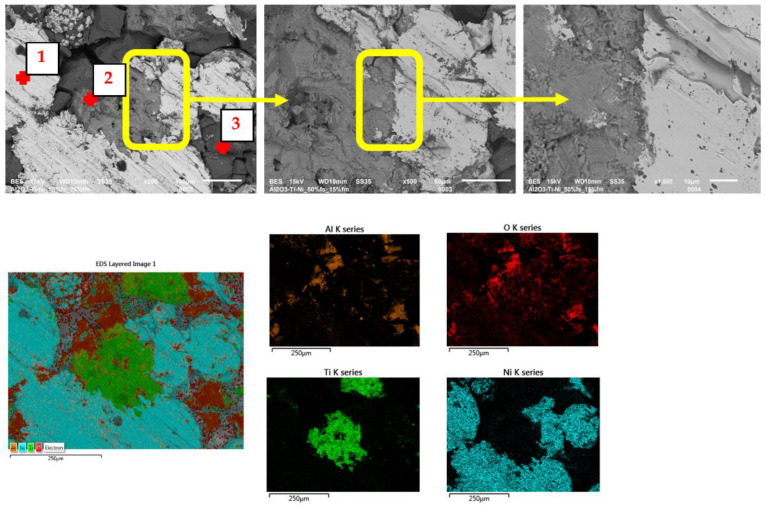
SEM/EDS results of samples with 10% volume of the metallic phase.

**Figure 11:**
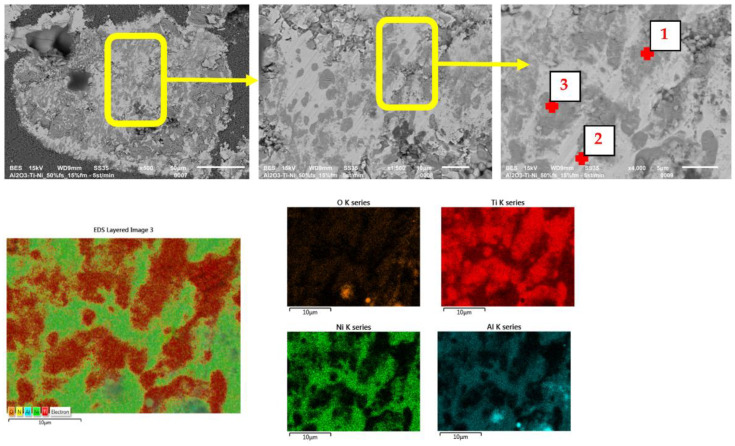
SEM/EDS results of samples with 15% volume of the metallic phase.

The SEM images (in the BSE mode—backscattered electrons) shown in Figure 9, Figure 10 and Figure 11 show the characteristic areas of sinters with different fractions of the metallic phase. In BSE mode, the alumina phase, titanium, nickel and solid solution appear as a different shade of gray: alumina appears as the darkest gray, nickel the lightest, while the solid solution is presented with intermediate gray color. Titanium at the figures appears as a lighter gray than alumina, and darker than solid solution. SEM tests revealed that the metallic phase particles are evenly distributed in the alumina matrix, no areas that are excessively enriched or depleted in the second phase were observed. The metallic phase is characterized by an irregular shape. Microstructure analysis confirmed that the metallic phase particles did not form agglomerates in the alumina matrix for all tested series of composites. Fractographic studies of the fractures showed that the main points of fracture initiation were the bonds between the ceramic matrix particles—Al_2_O_3_ and the metallic phase. Similar results were observed for all tested samples. Moreover, the observation of the cracks shows that the Al_2_O_3_ matrix was characterized by grain decohesion during cracking. In the next step, EDS X-ray spectroscopy was performed in order to obtain maps of the distribution of elements of the examined samples. The obtained maps of the distribution of elements are also shown in Figure 10a–c. The results of the chemical composition analysis are presented in Table 4. The results of the EDS analysis confirmed the chemical composition of the bright and dark areas in the SEM images as metals, solid solution and Al_2_O_3_. In all the tested samples, the presence of a solid solution of nickel and titanium, areas of pure titanium and nickel, and areas of the matrix were found. The XRD studies presented in Figure 8 identified the regions of the mixture of nickel and titanium as NiTi intermetallic phases.

## 4. Conclusions

The search for new solutions in the field of creating composite materials with unique properties has become the mainstream of current research. The aim of the research carried out in this study was to determine the possibility of producing ceramic-metal Al_2_O_3_/Ti/Ni composites by the slip casting method and to determine the influence of the metallic phase on the properties and microstructure of composites. A novelty in the proposed research is the possibility of adjusting the appropriate proportion of the metallic phase to the production of elements with a different chemical composition, including, in particular, the production of composites with the participation of intermetallic phases. Based on the obtained results, it can be concluded that the slurries proposed to produce composites exhibit shear thinning mechanism—their viscosity decreases with increasing shear rate. The lack of sedimentation tendency of the obtained suspensions proved their correct composition and the ability to produce composite samples from the Al_2_O_3_/Ti/Ni system. In all tested samples, before sintering, the presence of Al_2_O_3_, Ni and Ti peaks was revealed, while after sintering, the ceramic phase (Al_2_O_3_), metallic particles (Ni, Ti) and the NiTi intermetallic phase were observed. The structure of the composites is composed of an irregularly shaped metallic phase, which is uniformly distributed in the entire volume of the samples.

## Figures and Tables

**Table 1:** Specification of the powders used in the research based on the manufacturer’s data.

Material	Company	Average Particle Size	Density	Purity
α-Al_2_O_3_	Tamei Chemicals Co.	100 ± 25 nm	3.98 g/cm^3^	99.99%
Ni	Sigma-Aldrich	<50 µm	8.9 g/cm^3^	99.70%
Ti	GoodFellow Cambridge Limited	75 µm	4.51 g/cm^3^	99.50%

**Table 2:** Characteristics of the sample series included in the study.

No.	Chemical Composition	Solid Phase Content [%]	Metallic Phase Content [%]	Proportion of Ti to Ni in the Metallic Phase [%]
Series I	Al_2_O_3_/Ti/Ni	50%	5%	50/50
Series II			10%	
Series III			15%	

**Table 3:** Characteristics of the sample series included in the study.

Material	Theoretical Density [g/cm^3^] (Declared by the Manufacturer)	Real Density [g/cm^3^] (Pycnometric Method)
Al_2_O_3_	3.96	3.967 ± 0.028
Ni	8.90	8.7110 ± 0.045
Ti	4.51	4.5388 ± 0.0162

**Table 4:** Chemical composition of characteristic areas of the composites. Measurement areas are shown in Figure 9, Figure 10 and Figure 11.

Volume of the Metallic Phase	Point	Chemical Composition (%wt.)							
		O		Al		Ti		Ni	
		%wt.	%at.	%wt.	%at.	%wt.	%at.	%wt.	%at.
**5% (Figure 9)**	1	1.26	4.44	0.77	1.61	---	---	97.97	93.95
	2	33.54	59.28	3.64	3.82	61.07	36.06	1.75	0.84
	3	35.77	52.61	45.27	39.48	3.44	1.69	15.51	6.22
**10% (Figure 10)**	1	4.03	12.85	2.00	3.79	9.10	9.69	84.86	73.67
	2	10.57	26.99	11.64	17.62	8.26	7.04	69.53	48.35
	3	37.98	58.06	31.53	28.58	7.05	3.60	23.44	9.76
**15% (Figure 11)**	1	20.32	43.29	2.99	3.77	64.11	45.63	12.59	7.31
	2	---	---	13.56	24.20	26.43	26.57	60.02	49.23
	3	19.71	42.44	3.30	4.22	61.49	44.24	15.50	9.10

## Data Availability

Not applicable.
